# Comparative effects of 6-weeks progressive resistance exercise and moderate intensity aerobic exercise on CD4 count and weights of people living with HIV/AIDS in Alex-Ekwueme Federal University Teaching Hospital Ebonyi State

**DOI:** 10.1097/MD.0000000000028468

**Published:** 2022-01-14

**Authors:** Eucharia Ijego Asogwa, Okechukwu Sunday Abonyi, Chinyere Ori Elom, Christian A. Oduma, Chukwudum Collins Umoke, Nwele Anamelechi Ogai, Chidebe Chijioke Uwaleke, Ignatius Obilom Nwimo

**Affiliations:** a Department of Physiotherapy, Evangel University, Ebonyi State, Nigeria; b Faculty of Education, Ebonyi State University, Nigeria; c Alex Ekwueme Federal University, Ndufu Alike, Ebonyi State, Nigeria.

**Keywords:** cluster of differentiation 4, human immunodeficiency virus/acquired immunodeficiency syndrome, moderate intensive aerobic exercise, people living with HIV/AIDS, progressive resistance exercise, weight

## Abstract

**Introduction:**

The most significant clinical challenges in people living with HIV/AIDS (PLWHA) are decline in CD4^+^ T helper cells and abnormal weight reduction. Consequently, PLWHA who are on Anti-Retroviral Therapy (ART) or Highly Active Anti-Retroviral Therapy (HAART) are usually predisposed to coronary artery diseases due to abnormal weight gain (lipohypertrophy), though with improved and unstable Cluster of Differentiation 4 (CD4) counts.

The main aim of this study was to compare the effects of 6 weeks progressive resistance exercise (PRE) and moderate intensity aerobic exercise (MIAE) on CD4 count, and weight of PLWHA in Alex-Ekwueme Federal University Teaching Hospital Ebonyi State.

**Method:**

The study adopted quasi experimental research design. The population of the study was 60 Human Immunodeficiency Virus/Acquired Immunodeficiency Syndrome patients that attended HIV clinics at Alex-Ekwueme Federal University Teaching Hospital Abakaliki (AE-FUTHA) which formed 58 sample sizes for this study.

Simple random sampling technique was adopted for the study and flow cytometry, Heel Densitometer (X-rite 331C), and Omron BF 400 were the instrument used for data collection of CD4 counts and weight respectively; and they are standardized hence not validated. Mean, standard deviation and Analysis of Co-variance (ANCOVA) were used to analyze the data obtained. The reliability coefficient obtained from the pilot study was 0.848 and 0.994 for CD4 and WEIGHT respectively.

**Results:**

The major findings revealed a significant difference after 6 weeks’ PRE and MIAE on CD4 and Weight of PLWHA.

**Conclusion:**

Health promotion policy maker's arm of the government and Healthcare givers should integrate and enforce the use of exercises either as a single regimen or combined exercises into the management plan of PLWHA for greater boost in absolute CD4 count bearing in mind that both have positive effects. Physiotherapist should be integrated in the managements of PLWHA for appropriate prescription and education of therapeutic exercises for body weight.

## 1 Introduction

The most significant clinical challenges in people living with HIV/AIDS (PLWHA) are decline in CD4^+^ T helper cells and abnormal reduction in weight. CD4^+^ T helper cell is the main immune system infection fighter that triggers the body's response to infections.^[1]^ The cells’ reduction weakens the immune system and exposes the body to opportunistic infections as well as wasting syndrome (lipoatrophy). Consequently, PLWHA who are on Anti-Retroviral Therapy (ART) or Highly Active Anti-Retroviral Therapy (HAART) are usually predisposed to coronary artery diseases due to abnormal weight gain (lipohypertrophy).^[4]^ Surprisingly, it was recorded that a short burst of physical exercise increases blood Cluster of Differentiation 4 (CD4) lymphocyte counts,^[2]^ which perhaps may be a shift in the number of cells in circulation pathways, reflecting in changes of blood CD4 lymphocyte numbers.^[3]^ Research suggests that HIV-infected individuals who are on antiretroviral drugs can get significant physiological and immunological benefits from both progressive resistance exercise (PRE) and moderate intensity aerobic exercise (MIAE) after some weeks of training especially if performed 3 times a week at moderate intensity^[4]^; presenting both exercises as forms of therapeutic measures used in healthcare for management and improvement of health conditions of patients.^[5]^ According to Kisner and Colby (2012), PRE is a system of dynamic resistance training in which a constant external load is applied to the contracting muscles by some mechanical means and gradually increased using repetitive maximum as a basis for adaptation. PRE equally burns up glycogen stored in the muscles without oxygen, thus is a type of anaerobic exercise.^[6]^ Moreover, PRE is considered safe and beneficial for PLWHA and is said to be effective in improving their muscle strength, immune system and body compositions.^[28]^ Meanwhile, MIAE or endurance exercise which is defined as a regimen in moderate intensity form, containing aerobic interventions like cycling, stair stepping, rowing, and walking; usually meant to promote significant effects in cardiac endurance, aerobic capacity and weight; measured by maximal oxygen consumption is equally beneficial to PLWHA especially those on ART/HAART.^[4,29,30]^

However, it was found in a study by Dolan et al^[7]^ that at the end of 16 weeks of supervised home-based aerobic and progressive resistance training regimen in women infected with human immunodeficiency virus, no significant difference was observed in CD4 cell count between groups. While the combined PRE and MIAE group showed a significant increase in muscular strength and cardiorespiratory fitness as well as a significant decrease in waist circumference when compared to the controls. Another study indicated that after the intervention program, a significant difference in CD4 cell counts was found between the 2 groups (P 1/4 0.01).^[8]^ With respect to mental health, the same study recorded significant improvement in all subscales including anxiety disorder, social function, depression and mental health's total score were observed in the exercise group compared to the control groups (*P* < .001). It was equally found in a study that there was significant improvement and significant increase in CD4 cell counts and TG respectively between pre- and post- tests while total cholesterol (TChol), high density lipoprotein (HDL) and low density lipoprotein showed insignificant difference.^[9]^ The significant improvement in CD4 cell counts found in the experimental group agrees with the findings of some past studies, and relatively at variance with others which reported stability or insignificant improvements.^[10]^ reported insignificant increase in CD4 cell counts of HIV infected persons after 12 weeks aerobic exercise. However, other studies like^[11,12–14]^ reported an increase in CD4 cell counts, while few like^[26,14]^ found stability in CD4 cells.^[15]^ in their study demonstrated more stable CD4 cell counts from baseline of −3% in the experimental participants, while the control group experienced significant reduction of −16% after 12 weeks.

Furthermore, the weight loss, physical and mental health condition of the experimental group was better than the control group^[16]^ found in a study that there was no significant difference between both groups. This finding however did not support that of^[25]^ in which PRE and MIAE witnessed a statistical significant increase in weight of both groups (PRE and MIAE). In addition, progressive resistive exercise and aerobic exercise is proven to be safe and could be beneficial for adults living with Human Immunodeficiency Virus/Acquired Immunodeficiency Syndrome (HIV/AIDS) in their weight increment.^[5]^ More so^[27]^ in their study reported that there exists no statistical significant difference for weight after participant undergone MIAE and PRE intervention. Concurrently, another study recorded no statistical significant difference for weight after participant undergone MIAE and PRE intervention.^[22]^ However, the finding is not in support of^[17]^ who reported in a study that both PRE and MIAE had a significant reduction in weight for PLWHA.

In fact, the multisystem clinical manifestations of PLWHA which includes gross energy shortage, platelets reduction,^[18]^ cardiovascular weakness,^[19]^ immune reduction,^[20,21]^ and sometimes resistivity of ART drugs have been very much recorded with complimentary effects on both CD4counts and body weight.^[22]^ This is quite disturbing and has motivated lots of scientific investigations in area of exercises. Presently, very little work has been done here locally, while Nigeria positions the second most noteworthy of individuals living with HIV/AIDS around the world.^[23]^ Shockingly, the impacts of MIAE and PRE on CD4 and Weight has no general scientific consensus yet, it is still under debate; while a few scientists announced an improvement in their review, others found an abatement after routine exercise program. Consequently, the requirement for more experiments for clarification on impacts of MIAE and PRE on CD4 counts and body weight of PLWHA.

## 2 Purpose of the study

The main purpose of this study is to compare the effects of progressive resistive exercises and MIAE on CD4 counts and Bone Mineral Density of PLWHA who are on ART/HAART (highly active anti-retroviral therapy) in Enugu State University Teaching Hospital. Specifically this study will determine:

1. The comparative effects of PRE and MIAE on CD4 counts of PLWHA.
2. The comparative effects of PRE and MIAE on WEIGHT of PLWHA.

### 2.1 Research questions

1. What are the comparative effects of PRE and MIAE on CD4 counts of PLWHA?
2. What are the comparative effects of PRE and MIAE on WEGHT of PLWHA?

### 2.2 Hypotheses

For this study, the following null hypothesis will be tested at alpha level of 0.05

**Ho**_**1:**_ There will be no significant difference after 6 weeks comparative effects of PRE and MIAE on CD4 counts of people living with HIV/AID.
**Ho**_**2**_: There will be no significant difference after 6 weeks comparative effects of PRE and MIAE on WEIGHT of people living with HIV/AID.

## 3 Method

This study adopted an experimental research design with an equivalent (randomized) pretest and posttest data, utilized to observe the response of the dependent variables (CD4 and Weight) of the treatment group (MIAE and PRE). The setting for this study was at Physiotherapy department of Alex-Ekwueme Federal University Teaching Hospital Abakaliki (AE-FUTHA) in Ebonyi State. This health facility is a teaching hospital in Abakaliki. It has about 2000 beds, and up to 20 departments. This hospital was chosen based on the information gotten from health record that they diagnose more than 20 HIV/AIDS patients weekly and has up to 25% of 0.8% population of those living with HIV/AIDS in Ebonyi state. These include all HIV/AIDS patients that attend HIV clinics at AE-FUTHA between December 2019 to February 2020. The target population was 60 volunteers who were on ART/HAART (anti-retroviral treatment /highly active anti-retroviral treatment) for not less than 24 months.

Forty participants on ART/HAART who were willing to participate and met the inclusion criteria were randomly assigned to the 2 groups (A: aerobic, B: progressive, C: control), using balloting by replacement.^[18]^ However, only 58 subjects completed the study due to drop out by 2 persons from the control group. Thus, the sample size became 58 for the study.

## 4 Selection criteria

**A. Inclusion Criteria:**

- Only male HIV/AIDS patients within the age range of 18 to 60 years and female between 18 to 50 years of age.
- Only HIV/AIDS patients that have started taking their ART/HAART for the duration of 24 months and above, prior to the study, and attend HIV/AIDS clinics.

**B. Exclusion Criteria:**

Patients that are not on Anti-Retroviral therapy/ Highly Active Anti-Retroviral Therapy

- up to this duration of 24 months and above prior to the study
- All subjects with previous history of cardiac and diabetic complications.
- All pregnant subjects to avoid interference on CD4 count and weight.

The following instruments were used for data collection in this study:

- Flowcytometery (PartecCyflow counter), Germany.
- Heel Densitometer (X-rite 331C) Germany.
- Omron BF400 weighing scale

These instruments (i.e., Flow cytometry, Fluorescence Activated Cell Sorting and Heel Densitometer [X-rite 331C]) are standard and used worldwide. Hence, it does not need to be validated.

The obtained data during trial testing was subjected to Pearson Product Moment Correlation Co-efficient and the results were 0.848 and 0.994 for CD4 and WEIGHT respectively. These are said to be reliable because they are greater or equal to 0.8 according to.^[24]^

## 5 Experimental procedure

The procedure for data collection in this research was assisted by 4 field assistants including; a Physiotherapist, a Radiologist, a Nurse, a laboratory Scientist and a medical doctor. The subjects were recruited at AE-FUTHA and informed consent was issued explaining the purpose, procedure, and relevance of the study before the onset of the intervention. The intervention was supervised by Research and Ethics Committee (REC) of Alex-Ekwueme Federal University Teaching Hospital Abakaliki. All the willing participants were assessed for baseline data, which included age, weight, blood pressure (BP) and heart rate. Randomized control trial technique by balloting was used to divide the willing participants who met the inclusion criteria into 3 groups (A: aerobic, B: progressive and C: control). None of the groups were blind. The control group was not allowed to participate in the exercise and they were also asked not do any active exercise program for a period of 6 weeks of the study. The activities of daily living of the participants in the control group were monitored through a checklist and it was confirmed that none of them participated in any form of active exercise program. The MIAE and PRE (duration and frequency) of group (A) and (B) respectively was divided in this order: 18 sessions of sub-maximal aerobic exercises on Marshal Fitness bicycle ergometer and PA Pro Acting treadmill which consists of 4 stages:

1. 3 minutes warm up
2. 3 to 4 minutes to reach the Target Heart Rate (THR)
3. 20 minutes holding of the range of the THR, and
4. 3 minutes cool down for 3 times per week.

The intensity of the aerobic exercises were increased gradually in this order: 45% to 50% heart rate reserve during the first 2 weeks, 50% to 55% heart rate reserve during the second 2 weeks and 55% to 60% heart rate reserve during the last 2 weeks.

### 5.1 CD4 counts

Blood samples were drawn venopunctually using syringe into a test-tube. Reagents used were brought to room temperature, 850 μL of the count check bead green analyzed to make sure that the cyflow machine was working effectively. The needed numbers of Rohren test tubes were labelled appropriately and placed in a test tube rack. 20 μL of CD4 easy count kits (CD4 Mab-PE) were pipetted into them for the assay. Thereafter, 20 μL of blood samples were also pipetted into each test tube and incubated in the dark for 15 minutes at room temperature after mixing properly followed by the addition of 850 μL easy count. Lyse buffer was not added to each test tube. In order to avoid air bubbles, this was mixed properly and analyzed on the PartecCyflow. The outcome was displayed and copied from the screen.

### 5.2 Weight

The steps in data collection with Omron BF400 weighing scale started by setting the machine to display kilograms by adjusting the switch at the base, followed by placing it on the tile floor. The center of the scale was then pressed lightly with the researcher's foot to turn on the scale. The subjects were then asked to step onto the scale without their foot wears and heavy objects on them, placing their both feet side-by side on the feet prints land mark. However, the readings were displayed on screen and the records taken with the subject still standing still and upright on the scale.

### 5.3 Ethical consideration

Ethical approval was sought and obtained from the Research and Ethics Committee (REC) of Alex-Ekwueme Federal University Teaching Hospital Abakaliki with Registration Number AE-FUTHA/REC/VOL 3/2020/008. Participants’ privacy and confidentiality were maintained using code numbers instead of names, and ensured that records were destroyed at the end of the study. Subjects’ informed consent were obtained from the subjects before commencing the study and the principle of the Nuremberg declarations code^[20]^ on the protection of the right of subjects while conducting human experimental research was strictly observed.

## 6 Method of data analysis

The data obtained was analyzed using Statistical Package for Social Sciences (SPSS) version 25. The statistical tools used were mean, standard deviation, and analysis of covariance (ANCOVA).

## 7 Data analysis, presentation, and interpretations of result

**Research Question 1:** What is the comparative effect of PRE and MIAE on CD4 count of PLWHA?

By using the mean difference, the result in the graph above shows that there is an ordinal interaction effect of MIAE and PRE on CD4 of PLWHA. However, MIAE is higher in ranking in the graph than that of PRE within the period of 6 weeks while that of Control group has the least estimated marginal means with differences of 65.85, 27.95, and −76.67 respectively

The result in the bar chart above shows that MIAE has more effect on CD4 counts than PRE while the Control group has the least estimated marginal means of CD4 of PLWHA.

### 7.1 Research question 2

What is the comparative effect of PRE and MIAE on Weight of PLWHA?

By using the mean difference, the result in the graph above shows that there is an ordinal interaction effect of PRE and MIAE on Weight of PLWHA. However, PRE is higher in ranking in the graph than that of MIAE within the period of 6 weeks while that of Control group is lower than PRE but higher than MIAE based on estimated marginal means with differences of 2.07, −0.9 and 0.49respectively.

The result in the bar chart above shows that PRE has more effect on Weight than MIAE. However, the Control group is higher than MIAE based on estimated marginal means of Weight of PLWHA.

**Hypothesis 1:** *There will be no significant difference after 6 weeks’ comparative effects of PRE and MIAE on CD4 counts of people living with HIV/AIDS*

The result in the above table is on the effect of PRE, MIAE and Control on CD4 counts of participants in this study. The table shows a probability value (significant value) of 0.000 for PRE and MIAE and control group. The significant value in the table above for groups is less than the alpha level of 0.05. This means that the hypothesis earlier stated is not accepted. Thus, there is a significant difference after 6 weeks’ PRE, MIAE and Control on CD4 counts of PLWHA. The result in the table also shows that there is a positively high effect size of PRE, MIAE and Control on CD4 counts when the experimental groups are compared with the control group in the study.

By checking for pairwise comparison, the result in the above table is on the effect of PRE, MIAE and Control comparison based on CD4 in this study. The table shows that MIAE pair with PRE shows no significant difference; while MIAE pair with Control shows significant difference. PRE pair with MIAE shows no significant difference; while PRE pair with Control shows significant difference. Finally, Control pair with MIAE, and Control pair with PRE all show significant difference based on their respective probability value in the table above. Thus, all these show that the result is not consistent with that of Tests of Between-Subjects Effects which shows in general that there is a significant difference after 6 weeks’ PRE and MIAE on CD4 counts of PLWHA.

**Hypothesis 2:** *There will be no significant difference after 6 weeks’ comparative effects of PRE and MIAE on WEIGHT of PLWHA*

The result in the above table is on the comparative effect of PRE and MIAE on WEIGHT of people living with HIV/AIDS. The table shows a probability value (significant value) of 0.001 for the Group (PRE, MIAE, and Control) on WEIGHT. The significant value from the table above is less than the alpha level of 0.05. The decision rule is that if the probability value (significant value) is less than the alpha level of 0.05, then the earlier stated null hypothesis will not be accepted. Thus, we did not accept the earlier stated null hypothesis. Therefore, there is a significant difference after 6 weeks’ comparative effects of PRE, MIAE and Control on WEIGHT of PLWHA. The result in the table shows that there is a low effect size of PRE, MIAE and Control on WEIGHT and it implies that the mean difference has only little importance.

By checking for pairwise comparison, the result in the above table is on the effect of PRE, Bone Mineral Density and Control comparison based on WEIGHT in this study. The table shows that MIAE pair with PRE, MIAE pair with Control, PRE pair with MIAE, PRE pair with Control, Control pair with MIAE and Control pair with PRE all show not significant based on their respective probability value in the table above which are all greater than 0.05 alpha level. Thus, all these show that the result is not in line with that of Tests of Between-Subjects Effects that there is a significant difference after 6 weeks’ PRE, MIAE, and Control on WEIGHT of PLWHA.

## 8 Discussion of findings

### 8.1 Comparative effect of progressive resistance exercise, moderate intensity aerobic exercise and control on CD4 count of people living with HIV/AIDS

**Figure 1:**
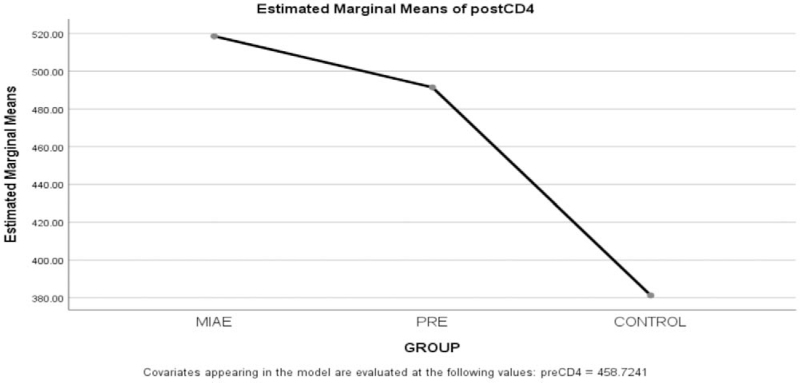
Comparative effect of Progressive Resistance Exercise and Moderate Intensity Aerobic Exercise on CD4 count of people living with HIV/AIDS.

**Figure 2:**
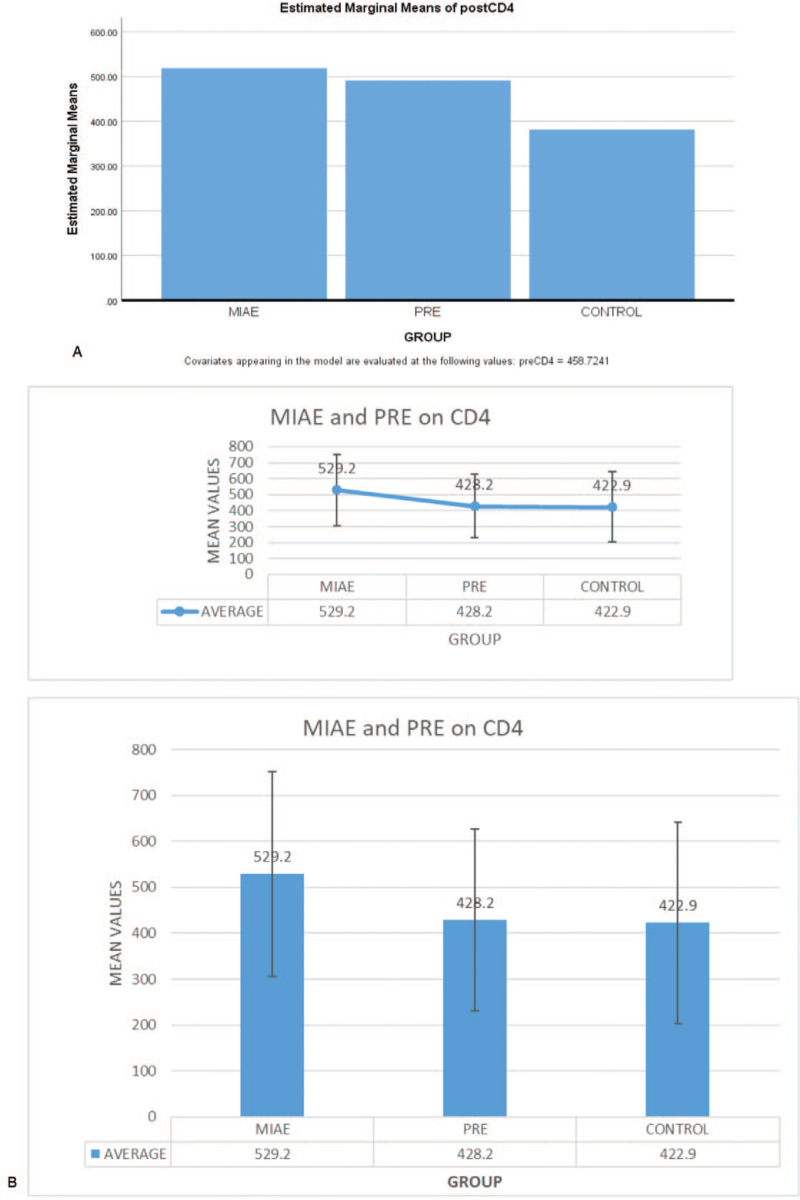
Estimated Marginal means of postCD4. Fig. 2b: Plots with error bars on Comparative effect of Progressive Resistance Exercise (PRE) and Moderate Intensity Aerobic Exercise (MIAE) on CD4 count of people living with HIV/AIDS.

Figure 1 show that the posttest is superior for PRE and MIAE but Control is lower than pretest. The MIAE is more superior to PRE by comparison while Control has the least. Thus, the mean difference effect of MIAE on CD4 counts is 65.85 while that of PRE on CD4 count is 27.95 implying that MIAE has more therapeutic increase compared to PRE. This result is in line with^[7]^ who recorded that PRE and MIAE had an increase in CD4 counts at the end of the interventions. This is also in line with^[9]^ who found in their study that there was a little increase in the CD4 counts of PLWHA after interventions. There is a significant difference after 6 weeks’ comparative effects of PRE and MIAE on CD4 counts of PLWHA. However, this finding does not support that of^[10]^ reported that MIAE and PRE have no significant effects on CD4 count on PLWHA (Fig. 2).

### 8.2 Comparative effect of progressive resistance exercise, moderate intensity aerobic exercise and control on WEIGHT of people living with HIV/AIDS

**Figure 3:**
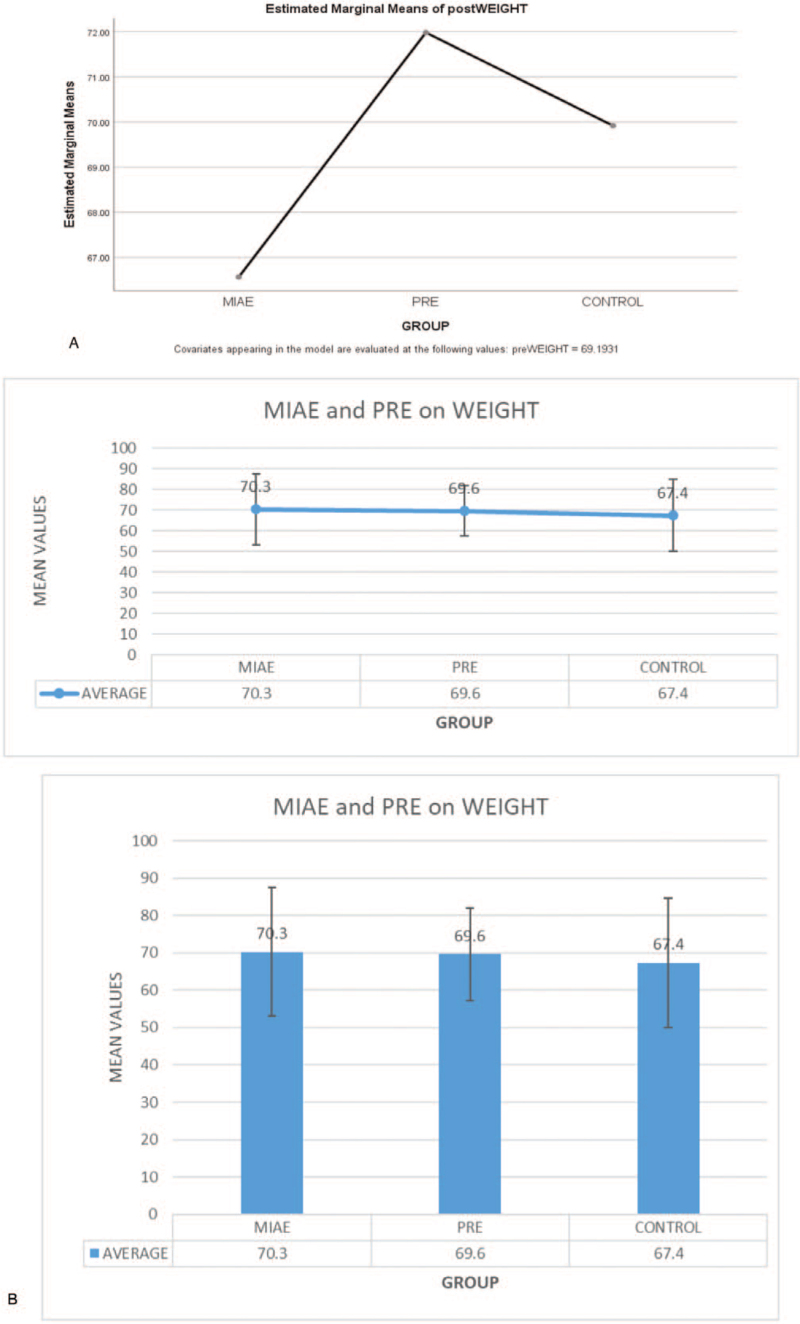
Comparative effect of Progressive Resistance Exercise (PRE) and Moderate Intensity Aerobic Exercise (MIAE) on Weight counts of people living with HIV/AIDS. Fig. 3b: Plots with Error Bar for Comparative effect of Progressive Resistance Exercise and Moderate Intensity Aerobic Exercise on Weight counts of people living with HIV/AIDS.

**Figure 4:**
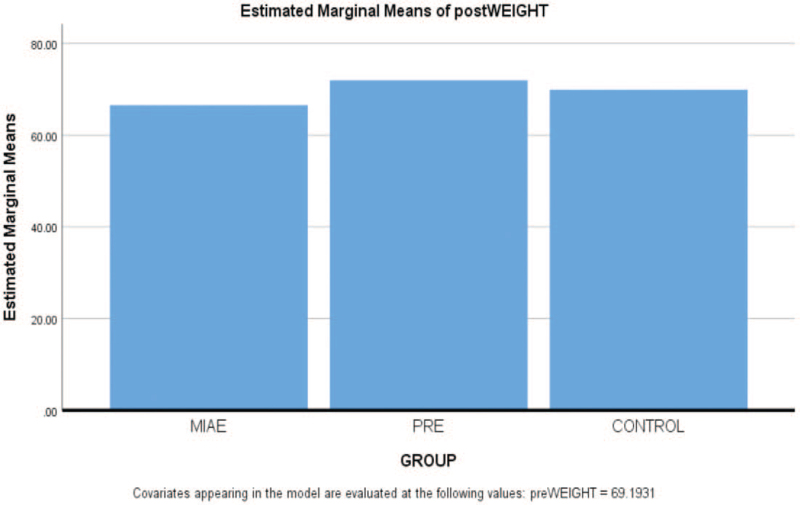
Estimated Marginal means of postWEIGHT.

Figure 3 shows that the PRE is higher than MIAE and Control is lower after 6 weeks of both exercises with the mean differences of 2.07, -0.9 and 0.49 respectively. Though, the finding is in line with^[25]^ who noted that both Progressive resistive and aerobic exercises increased body weight. In addition, progressive resistive exercise and aerobic exercise is proven to be safe and could be beneficial for adults living with HIV/AIDS in their weight increment.^[5]^ Therefore, there is a significant difference after 6 weeks’ comparative effects of PRE, MIAE and Control on WEIGHT of PLWHA. The finding of this study is not in agreement with^[27]^ who reported in their study carried out that there exists no statistical significant difference for weight after participant undergone MIAE and PRE intervention. However, the finding is in support of^[17]^ who reported in a study that both PRE and MIAE had a significant reduction in weight. Continuing, the author asserted that PRE and MIAE groups had significantly higher changes throughout the intervention in weight (Fig. 4).

## 9 Conclusion

The ordinal interaction in the graph which shows that MIAE had more effect on CD4 count compared to PRE, simply mean that MIAE group responded better in the production of CD4 cells. Moreover, the fact that there was net increase in both suggests that both exercises influenced the balance between T cell's formation and proliferation. It is possible that PRE might have stimulated more osteoblastic activity. The ordinal interaction in the graph which shows that PRE had more effect on WEIGHT compared to MIAE after weeks of both exercises simply seem to suggest that the PRE group responded better in weight gain. This might be as a result of greater influence PRE exacts on musculoskeletal system through loading and repetitive maximum leading to formation of greater muscle mass which in turn reflected in more weight gain than the MIAE group. On the other hand, the revealed significant difference of PRE and MIAE on CD4 counts of PLWHA suggested that both exercises have a differential effect on the dependent variable CD4 count. It equally implies that both of them were significantly more effective or beneficial than the other as far as production of CD4 T cells is concerned. The significant difference outcome of PRE and MIAE on WEIGHT of PLWHA after the duration of 6 weeks means that both exercises have differential effects on the dependent variable WEIGHT (Tables 1–6).

**Table 1:** Mean differences between moderate intensity aerobic exercise and progressive resistance exercise on CD4 after 6 weeks exercise.

Group	Pretest (Mean)	Posttest (Mean)	Differences in mean (Post-Pre)	Pretest (STD)	Posttest (STD)	Differences in STD (Post-Pre)
PRE	414.20	442.15	**27.95**	197.45	202.37	**4.92**
MIAE	500.95	566.80	**65.85**	225.25	212.47	**−12.78**
CONTROL	461.28	384.61	**−76.67**	237.41	199.20	**−0.40**

PRE = progressive resistance exercise, MIAE = moderate intensity aerobic exercise.

**Table 2:** Mean differences between moderate intensity aerobic exercise and progressive resistance exercise on weight after 6 weeks exercise.

Group	Pretest (Mean)	Posttest (Mean)	Differences in mean (Post-Pre)	Pretest (STD)	Posttest (STD)	Difference in STD (Post-Pre)
PRE	69.02	71.09	**2.07**	12.57	13.04	**0.47**
MIAE	70.83	69.93	**−0.9**	17.62	17.03	**−0.59**
CONTROL	67.03	67.57	**0.49**	17.84	17.15	**−0.69**

PRE = progressive resistance exercise, MIAE = moderate intensity aerobic exercise.

**Table 3:** Test of significant difference after 6 weeks’ comparative effects of progressive resistance exercise and moderate intensity aerobic exercise on CD4 counts of people living with HIV/AIDS.

Test between subject effects						
Dependent variable: postCD4						
Source	Type III sum of squares	Df	Mean square	F	Sig.	Effect size
Corrected Model	2167130.846^a^	3	722376.949	81.768	.000	.820
Intercept	70506.771	1	70506.771	7.981	.007	.129
GROUP^∗^ preCD4	2167130.846	3	722376.949	81.768	.000	.820
Error	477060.740	54	8834.458			
Total	15308302.000	58				
Corrected total	2644191.586	57				

a. R Squared = .820 (Adjusted R Squared = .810). b. Significant level-^∗^*P* < .05, Ns = Not significant: (*P* > .05). c. Effect size: d = 0.2 (small effect); d = 0.5 (medium effect); d = 0.8 (large effect). d. PRE = progressive resistance exercise, MIAE = moderate intensity aerobic exercise.

**Table 4:** Pairwise comparisons of progressive resistance exercise, moderate intensity aerobic exercise and control group of CD4 counts.

Pairwise comparisons dependent variable: postCD4						
					95% Confidence interval for difference^b^	
(I) GROUP	(J) GROUP	Mean difference (I-J)	Std. error	Sig.^b^	Lower bound	Upper bound
MIAE	PRE	27.124	27.814	.334	−28.640	82.889
	CONTROL	137.324^∗^	26.450	.000	84.295	190.353
PRE	MIAE	−27.124	27.814	.334	−82.889	28.640
	CONTROL	110.200^∗^	29.072	.000	51.914	168.486
CONTROL	MIAE	−137.324^∗^	26.450	.000	−190.353	−84.295
	PRE	−110.200^∗^	29.072	.000	−168.486	−51.914

Based on estimated marginal means. a. Significant level-∗*P* < .05, Ns = Not significant: (*P* > .05). b. Adjustment for multiple comparisons: Least Significant Difference (equivalent to no adjustments). PRE = progressive resistance exercise, MIAE = moderate intensity aerobic exercise.

**Table 5:** test of significant difference after 6 weeks’ comparative effects of progressive resistance exercise and moderate intensity aerobic exercise on WEIGHT of people living with HIV/AIDS.

Tests of between-subjects effects						
Dependent variable: postWEIGHT						
Source	Type III sum of squares	df	Mean Square	F	Sig.	Effect size
Corrected model	3483.620^a^	3	1161.207	6.017	.001	.251
Intercept	3666.739	1	3666.739	18.999	.000	.260
GROUP^∗^ preWEIGHT	3483.620	3	1161.207	6.017	.001	.251
Error	10421.582	54	192.992			
Total	293612.300	58				
Corrected total	13905.202	57				

a. R Squared = .251 (Adjusted R Squared = .209). a. Significant level-^∗^*P* < .05, Ns Not significant: (*P* > .05). b. Effect size: d = 0.2 (small effect); d = 0.5 (medium effect); d = 0.8 (large effect). c. PRE = progressive resistance exercise), MIAE = moderate intensity aerobic exercise.

**Table 6:** pairwise comparisons of progressive resistance exercise, moderate intensity aerobic exercise and control group on WEIGHT.

Pairwise comparisons						
Dependent variable: postWEIGHT						
					95% Confidence interval for difference^a^	
(I) GROUP	(J) GROUP	Mean difference (I-J)	Std. error	Sig.^a^	Lower bound	Upper bound
MIAE	PRE	−5.420	4.274	.210	−13.988	3.148
	CONTROL	−3.356	4.398	.449	−12.174	5.463
PRE	MIAE	5.420	4.274	.210	−3.148	13.988
	CONTROL	2.065	4.468	.646	−6.892	11.022
CONTROL	MIAE	3.356	4.398	.449	−5.463	12.174
	PRE	−2.065	4.468	.646	−11.022	6.892

Based on estimated marginal means. a. Significant level-^∗^*P* < .05, Ns = not significant: (*P* > .05). b. Adjustment for multiple comparisons: Least Significant Difference (equivalent to no adjustments). c. PRE = progressive resistance exercise, MIAE = moderate intensity aerobic exercise.

### 9.1 Limitations of the study

1. Refusal of many subjects to participate resulted to few number of subjects.
2. The economic situation of the country led to financial difficulties on both intervention group and the researchers because the study was self-sponsored the authors.

## 10 Educational implication of the study

1. The implication of research question one (1) finding is that if PLWHA with lower CD4 counts will participate more in MIAE other than PRE. This means that Exercise Physiologists and Health Managers will educate PLWHA to opt more for MIAE whenever they want to improve their CD4 counts.
2. The implication of research question 2 (2) results is that PLWHA who chose to take part in exercise for WEIGHT gain, will participate in PRE other than MIAE. Thus, the Exercise Physiologists and Health Managers will educate PLWHA and create awareness on the appropriate therapeutic exercise which in this case is PRE to help weight gain.

### 10.1 Recommendation

The followings are the recommendations based on the findings of the study:

1. Physical exercises especially MIAEs should be integrated as an adjunct therapy in the management of PLWHA who are either on Highly Active Anti-Retroviral Therapy for greater boost in immune system, resulting to improvement in absolute CD4 count.
2. Exercise Physiologists and Health Managers should educate and encourage PLWHA to engage in PRE which is the appropriate therapeutic exercise for weight gain.

## Author contributions

**Conceptualization:** Euharia Ijego Asogwa, Chinyere Ori Elom, Anamelechi Nwele Ogai, Chukwudum Collins Umoke.

**Data curation:** Okechukwu Sunday Abonyi, Chinyere Ori Elom, Christian Ajim Oduma, Chukwudum Collins Umoke, Chidebe Chijioke Uwaleke, Ignatius Obilor Nwimo.

**Formal analysis:** Euharia Ijego Asogwa, Okechukwu Sunday Abonyi, Chinyere Ori Elom, Anamelechi Nwele Ogai, Ignatius Obilor Nwimo.

**Funding acquisition:** Christian Ajim Oduma, Anamelechi Nwele Ogai, Chidebe Chijioke Uwaleke, Ignatius Obilor Nwimo.

**Investigation:** Euharia Ijego Asogwa, Chinyere Ori Elom, Chukwudum Collins Umoke, Chidebe Chijioke Uwaleke.

**Methodology:** Okechukwu Sunday Abonyi, Chinyere Ori Elom.

**Project administration:** Euharia Ijego Asogwa.

**Resources:** Christian Ajim Oduma, Anamelechi Nwele Ogai.

**Software:** Euharia Ijego Asogwa, Okechukwu Sunday Abonyi, Ignatius Obilor Nwimo.

**Supervision:** Okechukwu Sunday Abonyi.

**Validation:** Chinyere Ori Elom, Chukwudum Collins Umoke.

**Visualization:** Euharia Ijego Asogwa.

**Writing – original draft:** Euharia Ijego Asogwa, Okechukwu Sunday Abonyi, Anamelechi Nwele Ogai.

**Writing – review & editing:** Okechukwu Sunday Abonyi, Chinyere Ori Elom.
